# Integrating binary traits with quantitative phenotypes for association mapping of multivariate phenotypes

**DOI:** 10.1186/1753-6561-5-S9-S73

**Published:** 2011-11-29

**Authors:** Indranil Mukhopadhyay, Sujayam Saha, Saurabh Ghosh

**Affiliations:** 1 Human Genetics Unit, Indian Statistical Institute, 203 Barrackpore Trunk Road, Kolkata 700108, India

## Abstract

Clinical binary end-point traits are often governed by quantitative precursors. Hence it may be a prudent strategy to analyze a clinical end-point trait by considering a multivariate phenotype vector, possibly including both quantitative and qualitative phenotypes. A major statistical challenge lies in integrating the constituent phenotypes into a reduced univariate phenotype for association analyses. We assess the performances of certain reduced phenotypes using analysis of variance and a model-free quantile-based approach. We find that analysis of variance is more powerful than the quantile-based approach in detecting association, particularly for rare variants. We also find that using a principal component of the quantitative phenotypes and the residual of a logistic regression of the binary phenotype on the quantitative phenotypes may be an optimal method for integrating a binary phenotype with quantitative phenotypes to define a reduced univariate phenotype.

## Background

Clinical end-point traits are usually binary (affected/unaffected) in nature. However, these end points are often governed by quantitative precursors. On the other hand, a single quantitative trait may not be a sufficiently good surrogate for the end-point trait, and it may be more optimal to analyze a genetically relevant multivariate phenotype vector that includes both quantitative and qualitative phenotypes. Association analyses of multivariate phenotypes involve multiple statistical challenges, the primary one being the construction of the phenotype, particularly in the presence of both quantitative and binary traits in the multivariate phenotype vector. We assess the performances of some genetic association methods for certain choices of the multivariate phenotype vector using the data on the simulated phenotypes in the framework of the 1000 Genomes Project provided in Genetic Analysis Workshop 17 (GAW17).

## Methods

### Data description

For our analyses, we used the GAW17 data on the three quantitative traits (Q1, Q2, Q4) and the single binary trait for 697 individuals along with their genotypes at all the available 24,487 single-nucleotide polymorphisms (SNPs) distributed over the 22 autosomal chromosomes. We used data on age, sex, and smoking status (defined as a binary variable) as covariates because these factors could be potential confounders in the association analyses. We did not remove any SNP based on its minor allele frequency (MAF) because one of our goals was to identify rare variants involved in the etiology of the phenotypes. We performed our analyses on all 200 available replicates in the GAW17 data set.

### Statistical methods

Likelihood-based methods, such as variance components [1,2], have been traditionally used for the association mapping of multivariate phenotypes. However, such analyses are susceptible to the choice of the joint probability distribution of the components of the vector. In particular, the popular choice of a multivariate normal distribution for the vector of phenotypes is clearly inappropriate if even one of the components of the vector is binary in nature. Other methods [3,4] combine the association statistics of the different components (both binary and quantitative) of a multivariate phenotype vector but use a multivariate normality assumption for the vector of univariate statistics, thereby compromising the robustness of the method. An alternative approach that circumvents the problem of modeling the multivariate phenotype is to obtain a reduced univariate phenotype using principal components [5].

We define five univariate phenotypes based on the three quantitative traits Q1, Q2, and Q4 and the single binary trait (denoted *Z*): (1) the first principal component of Q1, Q2, Q4, and *Z* (denoted *T*_1_); (2) the first principal component of Q1, Q2, and Q4 only (denoted *T*_2_); (3) the first principal component of Q1, Q2, and Q4 with *Z* as a covariate (denoted *T*_3_); (4) a risk score of *Z* using Q1, Q2, and Q4 as predictors (denoted *T*_4_); and (5) the first principal component of Q1, Q2, Q4, and the proportion of the risk score of the binary trait unexplained by Q1, Q2, and Q4 (denoted *T*_5_). The principal components are computed on the basis of the variance-covariance matrix of the phenotypes included in the multivariate phenotype vector. The pairwise correlations (averaged over the replicates) between the different traits are as follows: (Q1, Q2): 0.23; (Q1, Q4): −0.31; (Q1, *Z*): 0.55; (Q2, Q4): 0.01; (Q2, *Z*): 0.4; and (Q4, *Z*): −0.53. The risk score is defined as the conditional probability of an individual being affected with respect to the binary trait (*Z* = 1) given the trait values (*X*_1_, *X*_2_, and *X*_3_) corresponding to the three quantitative phenotypes Q1, Q2, and Q4, respectively, and is computed using a binary logistic model:(1)

where the parameters *β*_0_, *β*_1_, *β*_2_, and *β*_3_ are estimated using the maximum-likelihood method. The proportion of the risk score unexplained by the quantitative traits is defined as  and is computed as the residual of the logistic regression of *Z* on *X*_1_, *X*_2_, and *X*_3_.

For the test of association, we assess the relative performances of two association methods on the reduced phenotype; the two methods are analysis of variance (ANOVA) and a modification of a novel quantile-based approach developed by our group [6]. The ANOVA tests for equality of means of the quantitative trait values across the three genotypic groups at a SNP, and the test statistic is distributed as *F*_2,694_ under the null hypothesis of no association. However, studies have shown that the assumption of homoskedasticity of the quantitative trait values for the different genotypic groups at a SNP, which is a requirement of ANOVA, may not be valid and may lead to misleading inferences on association [7,8].

The quantile-based regression approach is a model-free alternative that tests for equality of marker allele frequencies within different quantile intervals of the quantitative trait; it is based on the Armitage trend test [9]. We note here that the original quantile-based method [6] was based on a fixed number of quantiles. In addition, the test statistic in the original method was defined in terms of the slope coefficient of the linear regression of the frequencies of a marker allele on the mean quantitative trait values in the different quantile intervals; the regression coefficient is 0 under the null hypothesis of no allelic association However, our independent simulations showed that for SNPs with rare variants, the variation in estimated allele frequencies in most quantile intervals is minimal, and hence the estimated slope coefficient of the linear regression does not depart significantly from 0, resulting in reduced power.

In our modified approach, we compute the ratio of the between-quantile variance to the within-quantile variance of the proportion of allele frequencies along the lines of the ANOVA statistic for different numbers of quantile intervals ranging from 2 to 10. The optimal number of quantile intervals is determined by the maximum of this ratio, and the Armitage trend test is performed based on this number. Unlike the original regression test statistic, which depends on the number of fixed quantiles, the Armitage trend test statistic is distributed as a chi-square with 1 degree of freedom, irrespective of the optimal number of quantiles.

To correct for multiple testing, we use the false discovery rate (FDR) procedure [10] with an overall rate of 0.05 to identify SNPs significantly associated with the phenotype.

## Results

The GAW17 simulation model was available to us. To assess the power of detecting association, we considered the causative SNPs that modulated any of the component phenotypes in the multivariate phenotype vector. The empirical power at each such SNP was obtained as the proportion of replications in which the SNP was significantly associated with the relevant phenotype definition. Both the ANOVA and the quantile-based regression were adjusted for age, sex, and smoking status. Although the ANOVA approach incorporated these variables as covariates at the individual level, the quantile-based regression incorporated the mean values of these variables within each quantile interval as covariates. In Table 1 we present a comparison of the number of causative SNPs modulating Q1 and Q2 identified with empirical power 0.3, given an overall FDR of 0.05 for the different phenotype definitions.

**Table 1:** Number of causative SNPs identified by the two methods based on the five phenotype definitions with empirical power greater than 0.3

Actual phenotype	ANOVA		Quantile method	
	
	Q1	Q2	Q1	Q2
*T*_1_	15	7	11	3
*T*_2_	6	1	3	0
*T*_3_	4	1	3	1
*T*_4_	11	1	1	0
*T*_5_	18	12	12	8

We found that ANOVA identified more causative SNPs than the quantile-based method did. *T*_5_ yielded the maximum power and *T*_4_ the minimum power among the five phenotype definitions for both the ANOVA and the quantile-based method. We also found that *T*_2_ (which does not involve the binary trait *Z*) performed better than *T*_3_ (which uses *Z* as a covariate) with respect to the causative SNPs modulating Q1 or Q2. The maximum empirical power was obtained for the SNP C13S523 in the *FLT1* gene on chromosome 13 (modulating Q1) for all five phenotypes (1.0 for *T*_1_, *T*_2_, and *T*_5_, 0.99 for *T*_3_, and 0.89 for *T*_4_ using ANOVA; 1.0 for *T*_5_, 0.89 for *T*_1_, 0.82 for *T*_2_, 0.67 for *T*_4_, and 0.63 for *T*_3_ for the quantile-based method). The two flanking SNPs C13S522 and C13S524 (modulating Q1) also exhibited significant evidence of association based on *T*_1_ and *T*_5_ in a large proportion of replications.

The empirical powers corresponding to SNP C6S5380 in the *VNN1* gene on chromosome 6 (modulating Q2) were greater than 0.5 based on *T*_1_ and *T*_5_ and greater than 0.4 based on *T*_2_ and *T*_3_. Similarly, the SNPs C4S1877, C4S1878, and C4S1889 in the gene *KDR* on chromosome 4 (modulating Q1) were significantly associated based on *T*_2_ and *T*_5_ in more than 50% of the replications using ANOVA and in more than 40% of the replications based on *T*_1_ using ANOVA and based on *T*_1_, *T*_2_, *T*_3_, and *T*_5_ using the quantile-based method. All the significant SNPs mentioned have common variants (MAF > 0.01), except for C4S1877 and C4S1889, which have rare variants (MAF = 0.0007).

We also obtained significant evidence of association in more than 30% of the replications at C18S2492 in the gene *P1K3C3* on chromosome 18 with *T*_1_ and *T*_5_, and C17S4578 in the gene *PRKCA* on chromosome 17 with *T*_3_ (both modulating the latent liability trait and hence the binary trait *Z*).

## Discussion and conclusions

We have developed a method that integrates quantitative phenotypes with binary traits to construct a reduced univariate phenotype for association analysis of a multivariate phenotype vector. Among the five approaches to defining the reduced phenotype (labeled *T*_1_–*T*_5_) we found that both the model-based ANOVA and the model-free quantile-based regression yielded the maximum power for the phenotype defined by the principal components of the quantitative traits and the proportion of the risk score unexplained by the quantitative traits (*T*_5_) and the minimum power for the phenotype defined by the risk score of the binary trait as a function of the quantitative traits (*T*_4_). This finding can be explained by the fact that the risk score defined by *T*_4_ does not capture the complete information on variability of Q1 and Q2 and contains only that part of the variability that explains *Z*. Hence there is a loss of information and power when using *T*_4_ as an association phenotype. On the other hand, *T*_1_, which involves computation of principal components using the binary trait *Z* as a variable, is expected to capture less information on covariability of Q1, Q2, and Q4 compared to *T*_5_, which uses the continuous residuals of *Z* on Q1, Q2, and Q4 in the computation of the principal components. Because the binary trait *Z* is a function of Q1 and Q2, removing the effect of *Z* from a principal component involving Q1 and Q2 leads to a reduction in the information on both Q1 and Q2, and hence *T*_3_, which uses *Z* as a covariate, is less powerful than *T*_2_ (which does not involve *Z*) in identifying SNPs associated with Q1 or Q2.

The ANOVA approach provides consistently higher power than the quantile-based method. The ANOVA is a genotype-based method that models the distribution of quantitative phenotypes conditioned on genotypes. On the other hand, the quantile-based method is allele based and models the distribution of allele frequencies conditioned on phenotypic quantiles. If the sample size is reasonably large, then the asymptotic properties of the ANOVA statistic hold and the ANOVA is expected to be more powerful than the model-free quantile-based method. Although the modification of the original quantile-based method [6], which allows us to determine an optimal number of quantiles instead of fixing the number a priori, significantly increases the power to detect association, the Armitage trend test does not use the actual values of the quantiles and hence may contain less information on association than the ANOVA statistic does. We are currently exploring alternatives that can incorporate the quantile values into the association tests.

## Competing interests

The authors declare that there are no competing interests.

## Authors’ contributions

SG and IM developed the proposed method. SS wrote the computer codes and performed the data analyses. IM participated in the compilation and interpretation of the results. SG drafted the manuscript. All authors read and approved the final manuscript**.**
